# PERCEIVED NEEDS, FELT CONCERNS, AND QUALITY OF LIFE OF OLDER ADULTS IN MISSISSIPPI: AN INTERGENERATIONAL APPROACH

**DOI:** 10.1093/geroni/igac059.2047

**Published:** 2022-12-20

**Authors:** Muhammad Riaz, Laura Downey, Carolyn Adams-Price, Donna Peterson, Lori Staton, Alisha Hardman

**Affiliations:** Mississippi State University, Starkville, Mississippi, United States; Mississippi State University, Mississippi State, Mississippi, United States; Mississippi State University, STARKVILLE, Mississippi, United States; Mississippi State University, Mississippi State, Mississippi, United States; Mississippi State University, Mississippi State, Mississippi, United States; Mississippi State University, Mississippi State, Mississippi, United States

## Abstract

The purpose of the study was to identify felt needs and concerns of older adults living in their own homes in Mississippi using intergenerational perspectives. This mixed-methods study used snowball sampling to collect data through semi-structured interviews and structured questionnaires that asked about the quality of life and current and future problems among aging adults. Three generations of Mississippians participated in the study, including grandparents (N = 22), adult children (N = 23), and grandchildren (N = 19). Quantitative data were analyzed using SPSS, while qualitative data were managed with MaxQDA. Respondents identified concerns with physical health, difficulty living independently, and mental health problems affecting life satisfaction and quality of life. Incongruently with the intergenerational stake hypothesis, adult children were more invested in their children than their parents. Older adults who live closer to significant others and family were more resilient despite having physical and mental issues. Physical issues related to mobility, access to daily need services, and help with basic tasks like food preparation and mowing lawns, and issues related to mental health seemed less pronounced. Consistent with other research studies, older adults showed a positive sense of self. Similarly, parents showed resilience without being intrusive and overbearing while considering themselves as support for their children. Results could inform the development of programs or initiatives for grandparents, adult children, grandchildren, and others involved in caregiving activities and planning for older adults in Mississippi.

